# Demonstration by Infra-Red Imaging of a Temperature Control Defect in a Decompression Sickness Model Testing Minocycline

**DOI:** 10.3389/fphys.2019.00933

**Published:** 2019-07-24

**Authors:** Anne-Virginie Desruelle, Pierre Louge, Simone Richard, Jean-Eric Blatteau, Sandrine Gaillard, Sébastien De Maistre, Hélène David, Jean-Jacques Risso, Nicolas Vallée

**Affiliations:** ^1^Unité Environnements Extrêmes, Département Environnement Opérationnel, Institut de Recherche Biomédicale des Armées, Equipe Résidante de Recherche Subaquatique Opérationnelle, Toulon, France; ^2^Service de Médecine Hyperbare et Expertise Plongée, Hôpital d’Instruction des Armées, Toulon, France; ^3^Biotech Service, Université de Toulon, Toulon, France; ^4^Apricot Inhalotherapeutics, Saint-Laurent-de-l’Île-d’Orléans, QC, Canada

**Keywords:** ischemia, bubble, dive, neuroprotection, capillary leak, tetracycline, antibiotic treatment, extreme sports

## Abstract

The prevention, prognosis and resolution of decompression sickness (DCS) are not satisfactory. The etiology of DCS has highlighted thrombotic and inflammatory phenomena that could cause severe neurological disorders or even death. Given the immunomodulatory effects described for minocycline, an antibiotic in widespread use, we have decided to explore its effects in an experimental model for decompression sickness. 40 control mice (Ctrl) and 40 mice treated orally with 90 mg/kg of minocycline (MINO) were subjected to a protocol in a hyperbaric chamber, compressed with air. The purpose was to mimic a scuba dive to a depth of 90 msw and its pathogenic decompression phase. Clinical examinations and blood counts were conducted after the return to the surface. For the first time they were completed by a simple infrared (IR) imaging technique in order to assess feasibility and its clinical advantage in differentiating the sick mice (DCS) from the healthy mice (NoDCS). In this tudy, exposure to the hyperbaric protocol provoked a reduction in the number of circulating leukocytes. DCS in mice, manifesting itself by paralysis or convulsion for example, is also associated with a fall in platelets count. Cold areas ( < 25°C) were detected by IR in the hind paws and tail with significant differences (*p* < 0.05) between DCS and NoDCS. Severe hypothermia was also shown in the DCS mice. The ROC analysis of the thermograms has made it possible to determine that an average tail temperature below 27.5°C allows us to consider the animals to be suffering from DCS (OR = 8; AUC = 0.754, *p* = 0.0018). Minocycline modulates blood analysis and it seems to limit the mobilization of monocytes and granulocytes after the provocative dive. While a higher proportion of mice treated with minocycline experienced DCS symptoms, there is no significant difference. The infrared imaging has made it possible to show severe hypothermia. It suggests an modification of thermregulation in DCS animals. Surveillance by infrared camera is fast and it can aid the prognosis in the case of decompression sickness in mice.

## Introduction

Bubble formation following a decompression induces systemic inflammation and ischaemia, regularly linked to platelet activation (Pontier et al., 2011; Gempp et al., 2012), which can cause neurological disorders or even death (Brubakk et al., 2003; Vann et al., 2011).

In animals, we have recently suggested that preservation of immunological cross-talk between platelets and leukocytes (i.e., via αVβ3 and αMβ2) may be essential in preventing the occurrence of decompression sickness in its early phase, while inhibiting others (such as GPIIb/IIIa) would be helpful (Lambrechts et al., 2018). We have also shown the beneficial anti-inflammatory role of fluoxetine in DCS (Blatteau et al., 2015). Otherwise, a recent work underlined the fact that rapid decompression induced the phenotypic change from mononuclear phagocytes to M1 macrophages (Han et al., 2017). As our recent works tend to describe DCS as a sterile disease, we decided to use minocycline, commonly presented as a Disease Modifying Anti-Rheumatic Drug (Knuckley et al., 2008), to put forward these investigations concerning the impact of the immunological response in our mouse model of DCS.

Minocycline, a second-generation tetracycline, is mainly used for its anti-microbial activities but neuroprotective properties have also been described in recent years (Garrido-Mesa et al., 2013). Several studies have shown that minocycline exhibits anti-neurodegenerative properties (Amin et al., 1997; Whiteman and Halliwell, 1997). In an experimental model of cerebral ischaemia, minocycline inhibited microglial activation, reduced inflammation and decreased the size of an infarct (Yrjanheikki et al., 1999). Moreover, minocycline was able to inhibit caspase 1 and caspase 3 up-regulation and delays mortality (apoptosis) in traumatic brain injury (Sanchez Mejia et al., 2001) or in a mouse model of Huntington’s disease (Chen et al., 2000). In a model of autoimmune disease, research groups used the inhibitory effect of minocycline on microglia in order to promote neural stem cell re-activation (Rasmussen et al., 2011). Das et al. (2011) found similar results in a mouse model for encephalitis, and reproduced these results *in vitro* using microglia-conditioned media, while Ekdahl et al. (2009) observed similar effects for epilepsy. The findings of Rueger’s team were in good agreement with these results for treatment of stroke in the “chronic phase” and extended them with the first demonstration that this effect is directly attributable to minocycline itself, and not (only) indirectly mediated by microglia (Rueger et al., 2012).

Although minocycline is presented as protective or encouraging repair after trauma (Rueger et al., 2012; Garrido-Mesa et al., 2013; Drenger et al., 2018), it is possible that its preventive use interferes with the rapid reaction immune mechanisms required for cell protection or survival, in case of injury. Actually, minocycline was recently presented as promoting auto-immune disease (Irizarry-Caro et al., 2018). Minocycline inhibits caspase 1 (the proIL-1β-converting enzyme) (Sanchez Mejia et al., 2001), reduces cyclooxygenase-2 expression, prostaglandin E2 production (Yrjanheikki et al., 1999) and inducible nitric oxide synthase up-regulation (Yrjanheikki et al., 1998). Its use therefore inhibits immunological pathways including decreases in interleukin-1β and TNF-α (Afshari et al., 2018; Aparicio et al., 2018), or an increased expression ratio of Bcl-2/Bax and reduced expression of caspase-3 by the modulation of miR-155-mediated BDNF repression (Lu et al., 2018), an inflammatory and immune response regulator (Song and Lee, 2015). It was also suggested that it decreases the levels of toll-like receptor 2 (TLR2) content, and its adapter protein MyD88, as well as the levels of the protein NLRP3, which is indispensable in the composition of inflammasome (Garcez et al., 2018). Minocycline selectively inhibits M1 polarization (Kobayashi et al., 2013). Insofar as one can suspect the involvement of some of these pathways in the genesis of ADD, because of the polarization of macrophages (Han et al., 2017) or the likely role of TLR in DCS (Blatteau et al., 2018; Lambrechts et al., 2018) for example, it seems interesting to assess, at first in animals, the effects of minocycline, which still remains a drug widely prescribed in humans.

At the same time the nervous system, like the immune system or even the circulatory system, are known to regulate body temperature (Gordon, 2012, 2017). As these systems have been described as disrupted in DCS (Brubakk et al., 2003; Vann et al., 2011; Mazur et al., 2018), we wanted to verify using a simple method, i.e., infrared imaging, whether the temperature of the animals could also be disrupted. As Minocycline inhibited inducible nitric oxide synthase up-regulation (Yrjanheikki et al., 1998), we also verified whether it had an effect on the superficial temperature of the animals.

This study is the first to assess the effect of minocycline on the occurrence of decompression sickness in laboratory mice. On a mouse model treated with an acute dose of minocycline (90 mg/kg), we measured the clinical and biological effects of hyperbaric exposure causing DCS. We also completed this study by an infrared photography evaluation.

## Materials and Methods

**FIGURE 1:**
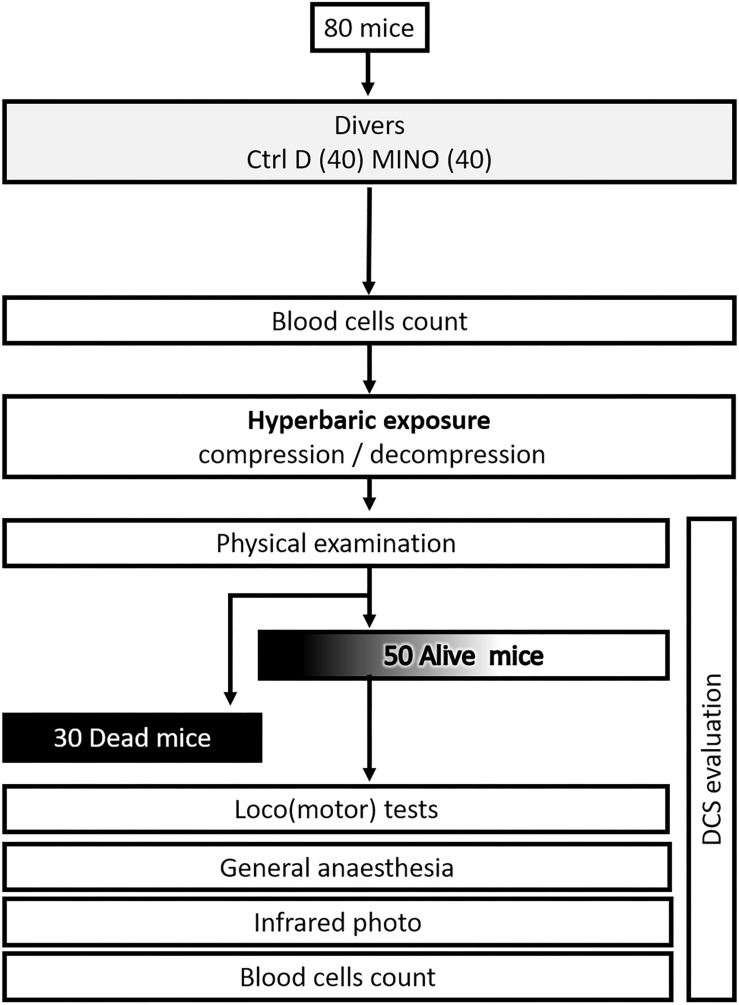
Flow chart process.

The experimental design can be followed in Figure 1.

### Animals and Ethics Statement

All procedures involving experimental animals were in line with European Union rules (Directive 2010/63/EU) and French law (Decree 2013/118). The Ethics Committee of the Institut de Recherche Biomédicale des Armées approved this study in 2016. According to our Animal Care Committee, a scoring system inspired by Swiss veterinary guidelines was implemented to ensure the welfare of animals. For each animal, a dedicated observer scored the stress or pain (from 0 to 3) relating to specific criteria listed on a form (see (Cosnard et al., 2017) for more information). Degree 3 pain (very painful) in one case or a total score of 12 in the table were the ethical endpoints. On this sheet, the most commonly found were: vocalizing, aggression or withdrawn behavior, reduction in exploratory behavior, licking, closed eyes, tears, bubbles in the eyes, high respiratory rate, runny nose, fur bristling, labored breathing, convulsions, paralysis, difficulty moving and problems with the fore or hind limbs (classified as motor disorders). In this study, no score reached 12 and there was no need to cull the animal based on these criteria. Actually, animals displaying Degree 3 convulsions died very rapidly. At the end of the experiment, mice were anesthetized first with halothane (5% in oxygen, Halothane, Belamont, France) in order to gain time and to minimize stress, and then with an intraperitoneal injection of a mixture of 16 mg/kg xylazine (Rompun^®^ 2%, Bayer Pharma) and 100 mg/kg ketamine (Imalgène^®^ 1000, Laboratoire Rhône).

Mice were housed in an accredited animal care facility. Mice were kept in cages (10 per cage) both during rest and during the experiments and maintained on a regular day (6:00 am–6:00 pm)/night (12 h) cycle. Food (kibble from Harlan Laboratories, 18% protein) and water were provided *ad libitum* and the temperature was kept at 22 ± 1°C.

The mice were separated into two groups: 40 control mice (Ctrl) and 40 mice treated with an acute dose of minocycline (Mino). Minocycline (Minocyne, Laboratoires Tonipharm Santé, 100 mg in capsule form) was administered by gavage to experimental animals as an oral solution 3 h before hyperbaric exposure, while the control group received a similar agarose minocycline-free solution. The microcrystals of minocycline were suspended so as to correspond to a dose of 90 mg/kg of minocycline in a 30% agarose-water solution. The dose was adjusted depending on the weight of the mice, corresponding to 2.7 mg of minocycline in 0.15 ml of solution for a 30 g mouse for example. This high dose of minocycline was determined on the basis of previous results from a mouse model of spinal cord ischaemia (Smith et al., 2013). They were 11–12 weeks’ old at the time of the hyperbaric protocol. There was no significant difference in their weight (KW_*Ctrl/Mino*_: *n* = 40/40, *p* = 0.919, Weight_*Ctrl*_ = 27.8; 3.2 g, Weight_*Mino*_ = 27.7; 2.5 g).

### Hyperbaric Exposure

Batches of 10 mice (10 per cage and 5 per group) from the Mino pool and the Ctrl pool were subjected to the hyperbaric protocol in a 200-l tank fitted with three observation ports. The mice were free to move around the cage.

The air compression protocol involved two ramps of pressure increase, first at 0.1 atm/min up to a relative pressure of 1 atm, followed by 1 atm/min up to 9 atm. 9 atm (equivalent to a depth of 90 msw) corresponds to the pressure at which animals were kept for 45 min before decompression. The decompression rate was 6 atm/min up to the surface. Compression and decompression were automatically controlled by a computer linked to an analog/digital converter (NIUSB-6211, National Instrument, United States) with two solenoid valves (Belino LR24A-SR, Switzerland) and a pressure transmitter (Pressure Transmitter 8314, Burket Fluid Control System, Germany). The software was programmed on a DasyLab (DasyLab National Instrument, United States) by our engineer. The software also monitored the temperature and oxygen level. Compressed air was generated using a diving compressor (Mini Verticus III, Bauer Comp, Germany) coupled to a 100-l tank at 300 bars. The oxygen analyser was based on a MicroFuel electrochemical cell (G18007 Teledyne Electronic Technologies/Analytical Instruments, United States). The temperature inside the tank was monitored using a platinum resistance temperature sensor (Pt 100, Eurotherm, France).

Water vapor and CO_2_ produced by the animals were captured with soda lime (< 300 ppm) and seccagel (relative humidity: 40–60%). Gasses were mixed by an electric fan. The day-night cycle was respected throughout (day at 0600/night at 1800).

### Behavior and Clinical Observations

At the end of decompression, a technician blinded to the group allocation observed mice in an open field over 30 min. The possible occurrence and the time to onset of the following manifestations were recorded: respiratory distress, moving difficulties, convulsions and death. Paralysis, difficulty moving and problems with fore or hind limbs were classified as motor disorders. A specific motor sensory test was also performed 30 min after the hyperbaric exposure. Grip tests – motor/sensory tests adapted from Hall (1985) – were used to quantify forelimb involvement 15 and 30 min after the end of decompression: the mouse is placed in the middle of a 60 cm long cord suspended at a height of 40 cm hanging from its front paws and its performance is timed over a period of 30 s maximum. Mice which escape by climbing up and then walking along the cord are given the highest score of 30 s. Mice which fail at least one test are considered to be symptomatic (direct fall or suspension time of less than 30 s). The results of this behavioral test are used to define clinical status and distinguish the following groups: mice that succumb from the provocative decompression (Fatal DCS), mice that failed at least one grip test and/or display clinical signs (DCS) and mice that passed the grip tests and display no clinical sign (No DCS).

### Anesthesia and Sacrifice

All mice were anesthetized 30 min after surfacing (after behavioral and clinical tests) first with isoflurane 4% (Isoflo, Axience SAS, France) to minimize stress, and then by intraperitoneal injection of a mixture of 16 mg/kg xylazine (Rompum^®^ 2%, Bayer Pharma, Germany), 100 mg/kg ketamine (Imalgene^®^ 1000, Rhône laboratory, France) and 1.65 mg/kg of acepromazine (Calmivet^®^, Vétoquinol, France).

Mice were kept for infrared evaluation, blood puncture, and then sacrificed by injection of pentobarbital (200 mg/kg i.p., Sanofi Santé, France).

### Evaluation of Decompression Sickness by Infrared Imaging

**FIGURE 2:**
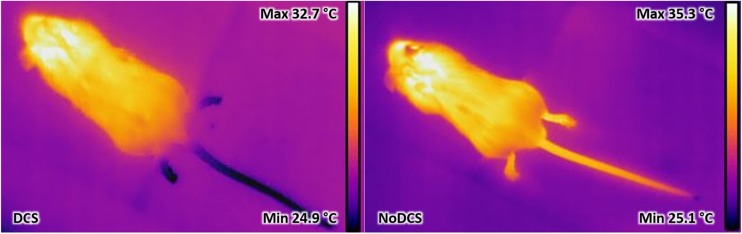
Example of infrared thermograms taken on mice C57Bl6 after leaving the hyperbaric chamber. The dark areas represent the coldest superficial areas and the light areas the hottest areas. In this example, the mouse on the left was considered during the clinical test to be suffering from decompression sickness. Dark areas were detected in the tail and its hind paws. The hottest area was generally not far from the ears. The mouse on the right was free from clinical signs indicating DCS. However, small cold area at the tip of the tail consecutive to the first blood sample taken before hyperbaric exposure was noted. It was also observed that in the tail the pilomotor reflex differs from the right-hand image: the swelling just before the beginning of the tail is larger on the right-hand image.

The surface temperatures of the mice were measured by infrared thermography (Figure 2). Thirty minutes after leaving the chamber, the mice were anesthetized and quickly placed on their stomachs under the thermal camera (Flir T62101 model, Sweden). Infrared pictures were made in a room at 23°C while the mice were placed on a polystyrene insulating surface covered with a surgical drape. The images were then analyzed using the processing software supplied with the camera (Flir ResearchIR). The highest value of the body surface temperature (Tmax) (revealing to be close to the head where the skin is directly visible, i.e., without fur), the average surface temperature of the tail ± SD, the percentages for the surface of the tail with a temperature lower than 27.5°C (see ROC analysis) and less than 25°C related to the total tail surface were calculated from these photos. The areas with a temperature lower than 25°C were also counted.

### Full Blood Analysis

Blood cell counts were analyzed in 15 μl samples taken from the tip of the tail and diluted in the same volume of 2 mM EDTA (Sigma, France). Blood tests were carried out using an automatic analyser (ABCvet, SCIL Animal Care Company, France) on samples taken 30 min before the dive and again 30 min afterward. In survivors, the second test values were corrected in accordance with the haematocrit (HCT) variation.

### Statistical Analyses

Individual blood cell count data were calculated as the percentage change from baseline (the measurement before hyperbaric exposure). Numerical data points were expressed as median and interquartile (median; interquartile). Different groups were compared using the Mann–Whitney (MW) test and paired comparisons within groups were analyzed using the Wilcoxon (W) test. Multiple comparisons were performed using the Kruskal–Wallis (KW) test followed by the Bonferroni–Dunn *post hoc* test. The significance threshold was 5% and *p*-values below this were assumed to indicate statistically significant differences. The optimal cut-off level for temperature which can discriminate between symptomatic or asymptomatic mice was determined using a Receiver Operating Characteristic (ROC) curve. The diagnostic value of this test was estimated through the calculation of sensitivity, specificity, negative predictive value and positive predictive value. With the calculation of the area under the ROC curve (AUC) and the two-tailed Fisher’s exact test was used to detect differences in the frequencies between symptomatic or asymptomatic animals from the temperature threshold; Odds Ratio (OR) and Likelihood Ratio (LR) with 95 percent confidence intervals (95% CI) were also calculated. The significance threshold was 95%.

## Results

### Clinical Status of the Animals Following the Dive

Following exposure to the hyperbaric protocol, on leaving the chamber some animals presented (KW_*Ctrl/Mino*_: *n* = 40/40, *p* = 0.869, 1stSymptom_*Ctrl*_ = 3.5; 6.6 min, 1stSymptom_*Mino*_ = 4.7; 7.4 min) neurological impairments with convulsions, paralysis, paresis or lameness. Respiratory problems were also observed with in particular dyspnoea, but no bleeding was observed in the sputum or nasal serosa (which eliminates problems of pulmonary barotrauma). In the most serious cases the animals were dead in 7.1; 4.8 min. In symptomatic animals, there was no significant difference in the time of appearance of the first symptoms, death or in their proportion of expression.

**FIGURE 3:**
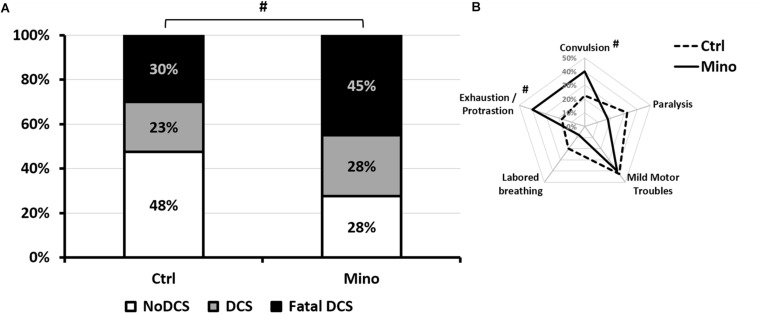
Symptoms in different groups of mice 30 min after pathogenic decompression as a function of drug treatment. **(A)** Histogram: The Fatal DCS status (Black blocks) encompasses mice that die in the 30 min following the end of the hyperbaric protocol. The DCS status (gray blocks) was attributed when the mice presented neurological signs in the form of paresis or paralysis of at least one limb, convulsions and/or reduced performance in the motor test for forelimbs. White blocks represent the proportion of mice that passed both grip tests and shown no clinical sign (NoDCS). **(B)** Radar Chart: percentage of mice displaying a type of symptom in a population, considering that a mouse can present several symptoms at the same time. Ctrl, Control mice (no active molecules); Mino, Minocycline (90 mg/kg), ^#^Denotes *p* < 0.10 (trend) between groups.

Treatment with minocycline only tended to increase symptoms linked to decompression (Figure 3) such as convulsions (KW_*Ctrl/Mino*_: *n* = 40/40, *p* = 0.093), and apparent fatigue (prostration and isolation from the group) (KW_*Ctrl/Mino*_: *n* = 40/40, *p* = 0.077) the effects of which were seen more globally in the status (KW_*Ctrl/Mino*_: *n* = 40/40, *p* = 0.072). The treatment had no significant impact on the other clinical signs.

### Full Blood Counts

#### Influence of the Hyperbaric Protocol

**FIGURE 4:**
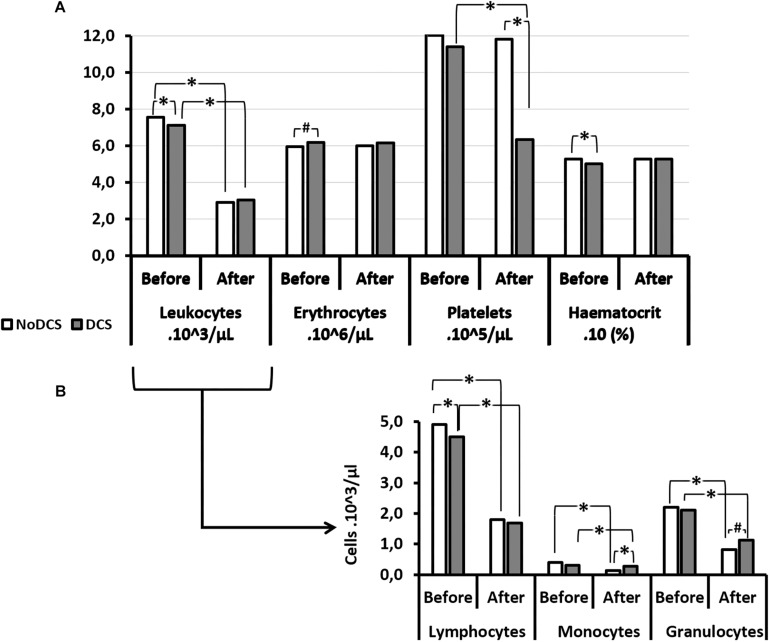
Blood cell count **(A)** and its leukocytes details **(B)** before and after the hyperbaric exposure, according to the clinical outcome. As results showed haemoconcentration linked to the hyperbaric exposure, the blood parameters registered after the dive were corrected individually depending on the variation in the haematocrit (notify by “c”), and then compared to the first blood punctures to present their changes (%): ^*^Notes significant difference (*p* < 0.05) and *^#^* denotes *p* < 0.10 (trend) between NoDCS and DCS groups for the same paramete. NoDCS, mice without symptom of decompression sickness (white blocks); DCS, mice displaying symptoms of decompression sickness (gray blocks).

Overall (all groups included) the number of circulating white blood cells (WBC), and the number of platelets (PLA), decreased significantly following the hyperbaric protocol (W: WBC_*Before*_ = 7.29; 2.14 10^3^/μl; WBC_*After*_: 2.99; 1.35 10^3^/μl; *p* < 0.0001; W: PLA_*Before*_ = 1155; 231 10^3^/μl; PLA_*After*_: 955; 539 10^3^/μl; *p* < 0.001). The number of red blood cells (RBC) and the haematocrit (HCT) did not vary (W: RBC_*Before*_ = 6.00; 1.19 10^6^/μl; RBC_*After*_: 6.12; 1.42 10^6^/μl; *p* = 0.763; W: HCT_*Before*_ = 52.6; 13.7; HCT_*After*_: 54.4; 19.3; *p* = 0.184) (Figure 4).

There were some interesting associations between certain factors and the clinical outcomes. Thus it was observed that the haematocrit value, even before having dived, was significantly lower (*p* = 0.024) in the subjects which will present a DCS. In it their red blood cells were smaller (MCV: *p* = 0.032) and there tended to be fewer of them (*p* = 0.058). Even though the total number of leukocytes did not seem to be determinant, it appeared that there were fewer circulating lymphocytes (*p* = 0.046) before the dive in mice with DCS after the dive.

After the dive and at the onset of the DCS, it was observed that the number of circulating platelets was significantly lower in the DCS mice (*p* < 0.0001), with de facto a higher magnitude of response (in the sense of a decrease) compared with the mice which did not present DCS. In parallel, the mean platelet volume (MPV) increased (*p* = 0.006) following the hyperbaric protocol.

As far as the leukocyte count after the dive was concerned, even though their overall number did not seem to vary according to the diagnosis, it appeared that the count of their sub-populations revealed disparities related to the clinical status of the mice. So in the NoDCS mice a significant reduction in the number of circulating monocytes post-dive (*p* = 0.004) was observed, obviously with a greater magnitude of variation (i.e., their mobilization) (*p* = 0.049). It could also be noted that these NoDCS animals tended to have fewer circulating granulocytes after the dive (*p* = 0.056). However, the magnitude of granulocyte mobilization did not seem to differ between the DCS and NoDCS animals (*p* = 0.488).

#### Influence of the Treatment

**FIGURE 5:**
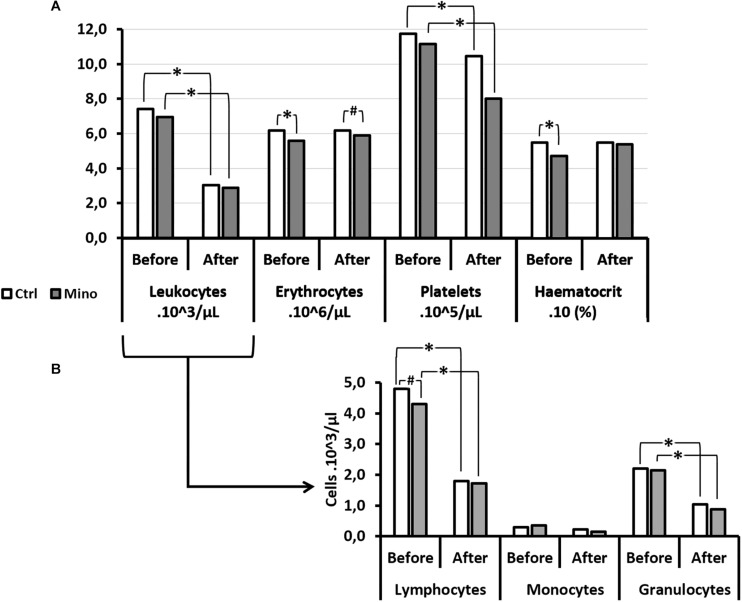
Effects of Minocycline on blood cells count before and after the hyperbaric exposure. **(A)** Histograms represent main blood parameters. **(B)** Histograms focused on white blood cells sub-populations. As results showed haemoconcentration linked to the hyperbaric exposure, the blood parameters registered after the dive were corrected individually depending on the variation in the haematocrit (notify by “c”). ^*^Notes significant difference (*p* < 0.05) and ^#^ denotes *p* < 0.10 (trend) between groups. Ctrl, Control mice (no active molecules, white blocks); Mino, Minocycline (90 mg/kg, gray blocks).

The treatment might have influenced some blood variables (Figure 5). However, there was no difference in the magnitude of the variations between the Mino and the Ctrl mice, which would have suggested that the treatment did not influence the body’s response to DCS.

A significant influence of the treatment was observed on the number (downward) of erythrocytes before the dive (*p* = 0.005) which tended to continue after the dive (*p* = 0.066), without there being any difference before and after the hyperbaric exposure. The haematocrit had also decreased (*p* = 0.001) with the treatment before the hyperbaric exposure. This observation was no longer true afterward. At the same time, the Mean Corpuscular Volume (MCV) (*p* < 0.001) and the Red Cell Distribution Width (RDW) (*p* = 0.012) decreased significantly in animals treated before diving, which overall would suggest plasmolysis of the erythrocytes before the dive. This difference between the groups treated was no longer observed after the dive.

A significantly higher mean platelet volume (MPV) was noted in the Mino mice (*p* = 0.001) following the treatment which continued after the dive (*p* = 0.008), however, without this affecting their number significantly either before or afterward.

**FIGURE 6:**
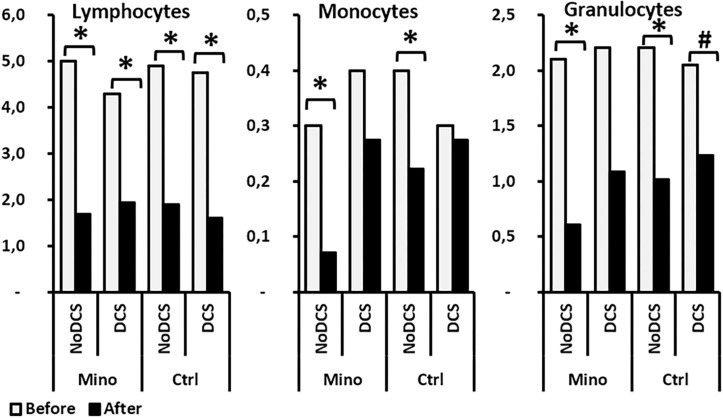
Effects of Minocycline on white blood cells count before and after the hyperbaric exposure, according to the clinical status and the treatment. As results showed haemoconcentration linked to the hyperbaric exposure, the blood parameters registered after the dive were corrected individually depending on the variation in the haematocrit. ^*^Notes significant difference (*p* < 0.05) between before and after the dive for the same parameter, and ^#^ denotes *p* < 0.10 (trend) between groups. Ctrl, Control mice (no active molecules, white blocks); Mino, Minocycline (90 mg/kg, gray blocks).

As far as the leukocyte count was concerned, even though their overall number did not seem to vary according to the treatment, it appeared that the count of their sub-populations revealed disparities (Figure 6). Therefore, in mice treated with minocycline, a tendency to had fewer circulating lymphocytes before the dive (*p* = 0.083) was observed. The absence of a significant difference between the monocyte counts that showed DCS was observed. However, the magnitude of granulocyte sub-population mobilization did not seem to differ between the Ctrl and Mino animals following the dive.

### Infrared Photographs

#### Influence of the Hyperbaric Protocol

The infrared images had made it possible to determine the animal’s maximum surface temperature (Tmax) or to distinguish the variations in hot areas such as the extremity of the hind paws or the tail. The animal’s tail has been the subject of a more in-depth thermal study insofar as it has relatively little hair.

**FIGURE 7:**
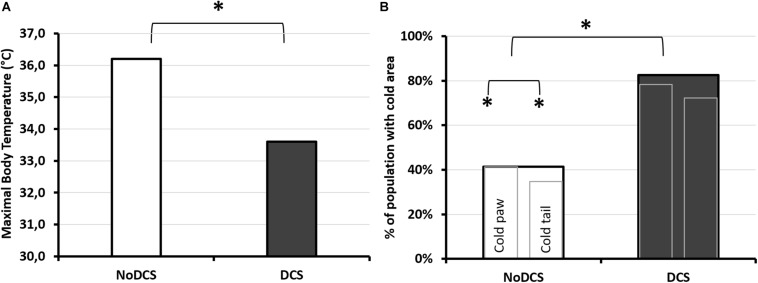
Infrared analysis depending on the clinical diagnosis following the pathogenic hyperbaric protocol. **(A)** Maximum body temperature observed by infrared photography. The photograph was taken 30 min after leaving the hyperbaric chamber, on animals under general anesthetic with isoflurane, lying on their stomachs. **(B)** Percentage of mice diagnosed with DCS (dark histogram) or asymptomatic (NoDCS, white histogram) presenting cold areas of less than 25°C detected by IR camera in the paws and/or the tail. Presence or absence of “cold” area in the animal. The largest histogram incorporates the two smaller ones. The left-hand one shows the percentage of mice with one or more cold areas in the hind paws (without differentiating right from left), and the right-hand one showing the percentage of mice with one or more cold areas in the tail. ^*^ marks a significant difference (*p* < 0.05) and ^#^ a tendency (*p* < 0.10). *n*_*NoDCS/DCS*_ = 29/23.

Processing of the infrared images has made it possible to show that the maximum surface temperature (Figure 7A) of the mouse’s body visible via the camera was lower in DCS mice than in NoDCS mice (Body T°max; T_*DCS*_ = 33.6; 2.0°C; T_*NoDCS*_ = 36.2; 0.9°C; KW: *p* < 0.0001). It also emerged that the proportion of mice presenting cold areas in the rear paws and/or the tail (Figure 7B) was higher in the DCS animals (Tail and paws: KW *p* = 0.003; Paws KW *p* = 0.019; Tail *p* = 0.001).

**FIGURE 8:**
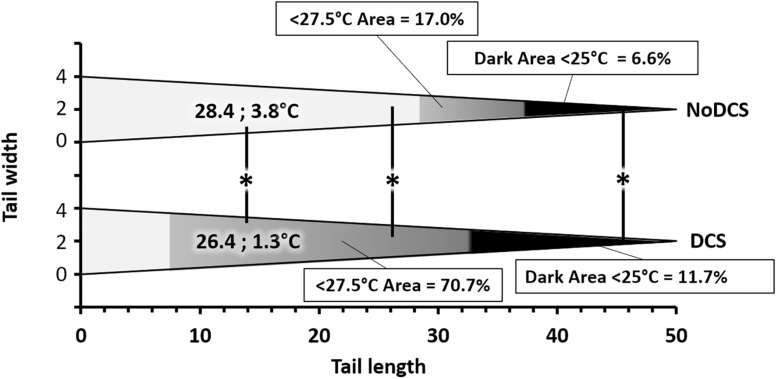
Relative surface area of thermal areas ( > 27.5 and > 25°C) in the tail of symptomatic (DCS) and asymptomatic (NoDCS) mice. The area lower than 27.5°C was determined by the ROC statistical method. It includes the area lower than 25°C. This graph was established for a standardized tail of the triangular type and a total surface of 100 pixels, i.e., 50 pixels in length over 4 pixels at its greatest width. The areas, starting at the tip, are expressed as a percentage of the total surface of the tail. The median superficial temperature of the tail is displayed directly on the graph. ^*^ marks a significant difference (*p* < 0.05).

The average superficial temperature of the tail of DCS mice, as well as its variability, were significantly lower than those of the NoDCS mice (T_*DCS*_ = 26.4; 1.3°C; T_*NoDCS*_ = 28.4; 3.8°C; KW: Mean *p* = 0.003, SD *p* = 0.002). With the use of ROC curve analysis (Figure 8) on data including mice whatever the treatment, with NoDCS being asymptomatic and therefore used as negative event, the threshold value of the mean temperature of the tail, predictive of DCS, was determined as < 27.5°C, with corresponding values of sensitivity and specificity as follows: 76% (95% CI, 54–89%), 71% (95% CI, 52–85%). Positive-predictive value and negative-predictive value were 67% and 80%, respectively. Odds Ratio (OR) and Likelihood Ratio (LR) values were as follows: OR = 8.00 (95% CI, 2.28–28.01), LR+ = 2.66 (95% CI, 1.41–5.02) and LR- = 0.33 (95% CI, 0.15–0.74). Area Under the Curve, explaining differences in the frequencies between symptomatic or asymptomatic, was 0.75 (95% CI, 0.59–0.91) and it was significantly different from 0.50 (Fisher test: *p* = 0.0018).

It also seemed that the surface area of the coldest area on the tail, appearing in black on the images and for which the temperature was less than 25°C, could had a link to the clinical status of the animals, insofar as the DCS animals (11.7; 31.1%) (*p* = 0.039) had a larger cold area at the tip of the tail than the NoDCS animals (6.6; 11.5%) (Figure 8).

After application of the temperature threshold determined by the ROC analysis it appeared that the surface area where the temperature was less than 27.5°C, could had a link to the clinical status of the animals, insofar as the DCS animals (70.7; 31.1%) (*p* = 0.006) had a larger cold area at the tip of the tail than the NoDCS animals (17.0; 58.5%) (Figure 8).

#### Influence of the Treatment

**FIGURE 9:**
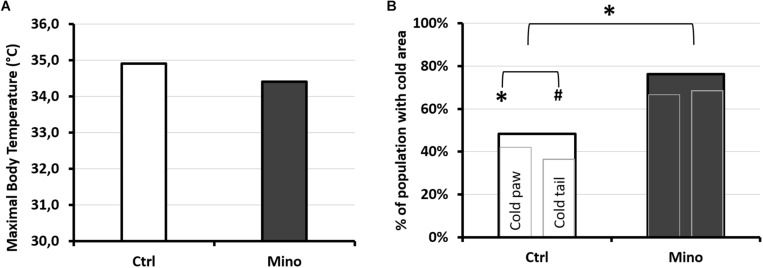
Infrared analysis depending on treatment after exposure to the protocol causing decompression sickness. **(A)** Maximum temperature observed by infrared photography. The photograph was taken 30 min after leaving the hyperbaric chamber, on animals under general anesthetic with isoflurane, lying on their stomachs. **(B)** Percentage of mice treated with minocycline (Mino, dark histogram) or control (Ctrl, white histogram) presenting cold areas detected by IR camera in the paws and/or the tail. Presence or absence of “cold” area in the animal. The largest histogram incorporates the two smaller ones. The left-hand one shows the percentage of mice with one or more cold areas in the hind paws (without differentiating right from left), and the right-hand one showing the percentage of mice with one or more cold areas in the tail. ^*^ marks a significant difference (*p* < 0.05) and # a tendency (*p* < 0.10). *n*_*Ctrl/Mino*_ = 31/21.

In the body, processing of the infrared images had not made it possible to show that the maximum superficial temperature, measured following the hyperbaric protocol, may have been influenced by the type of treatment (KW: *p* = 0.184) (Figure 9A). No significant difference was shown in the temperature of the tail depending on the treatment (T_*Ctrl*_ = 27.7; 3.1°C; T_*Mino*_ = 26.9; 3.7°C; KW: *p* = 0.935), or even in the expanse of the black cold areas (*p* = 0.929), or also in the cold areas determined by the ROC method (p = 0.680). However, it emerged that the proportion of mice presenting cold areas in the hind paws and/or the tail (Figure 9B) was higher in the Mino animals (Tail and/or paws: KW *p* = 0.047; Paws KW *p* = 0.043; Tail *p* = 0.083).

## Discussion

This dive protocol, as in previous studies (Blatteau et al., 2012; Vallee et al., 2012, 2016), was accompanied by a global reduction in the number of circulating leukocytes. It produced DCS, seen overall by an alteration in clinical and behavioral performances, particularly indicating neurological impairment, and also associated to a reduction in platelets count. Severe hypothermia was also seen in the animals suffering from DCS.

The minocycline dose was administered 4 h before the provocative decompression. Oral gavage was used to limit the complication of an additional treatment-induced inflammatory response from the sclerosing properties of minocycline when administered i.p. or subcutaneously (Light et al., 1994; Vonder Haar et al., 2014). The systemic (i.p.) dose of minocycline that is required to reach a brain concentration of about 36 μM was reported to be 90 mg/kg for mice (Milane et al., 2007), a dose that is associated with unpleasant side effects such as inflammatory reactions in the peritoneum (Fagan et al., 2004).

Within the framework of this experiment, we find ourselves in conditions close to the dose described as being effective for the brain, whilst preferring the oral route to avoid it being too toxic. This should result in systemic minocycline levels lower than 36 μM, although we did not directly measure its levels after the dive. In this study, a higher proportion of mice treated with minocycline 90 mg/kg suffered DCS, but this difference was statistically insignificant: from a clinical point of view, Mino mice manifested a slightly larger number of convulsions and prostration, and also a larger proportion of mice presenting cold areas whether at the tail or hind paws.

### Platelet and Leukocyte Mobilization Is Associated to the Clinical Profile

Platelet recruitment and leukocyte recruitment are present following a provocative dive. This has been regularly described in DCS, arguing for a syndrome that is both thrombotic and inflammatory. This pattern also seems to be emerging in these experiments, via the reduction in the number of circulating platelets and leukocytes after the dive. The platelet mobilization would be greater in DCS cases. However, subtleties concerning the level of monocytes and macrophages mobilization seem to emerge as a function of diagnosis and to a lesser extent of the effects of minocycline.

#### Red Blood Cells

Before the dive, the use of minocycline was associated with a decrease in the number of circulating red blood cells and their Mean Corpuscular Volume. This therefore affected the haematocrit. It could suggest minocycline-induced plasmolysis, probably due to a potassium leakage as previously described by Lannigan and Evans (1982). It is almost possible to attribute formally the decrease in the number of erythrocytes to minocycline (Lannigan and Evans, 1982). Even if it does not seem reasonable to say that the effect of minocycline on the red blood cells is directly related to the accidentology, it might be interesting to check if having a low haematocrit level, with in this case small RBCs, would be a predisposing element in the occurrence of DCS. In addition, as this is no longer true after the dive, it could suggest that older (i.e., less enlarged) red blood cells disappeared preferentially in DCS. It could also be suggested that new reticulocytes were released into circulation from the spleen, but Guerrero’s team refuted this theory (Lambrechts et al., 2014). It should be remembered that reticulocytes are slightly larger than mature erythrocytes so that, overall, a raised MCV may be due to a larger number of these immature red cells. In DCS, the most common theory refers to the destruction of cells by bubbles.

#### Platelets

Globally, the platelets are bigger on average after the dive. This can be seen as either a release of new platelets following salting-out by the spleen, but this latter hypothesis seems to be unfounded (Lambrechts et al., 2014), or by destruction of the oldest platelets (which are smaller) by bubbles or their mobilization in the context of a thrombotic or inflammatory cascade (Lambrechts et al., 2018). Although treatment with minocycline does not seem to affect platelet mobilization directly, it seems that the plasmolytic effect of minocycline results in the selection of a less mature platelet pool (with bigger platelets both before and after the dive). Hypothetically, it is possible to suggest that these platelets are less likely to react to surrounding stress. This would explain why the Mino mice do not see their platelet pool reduce more significantly than that of the Ctrl mice, despite more significant symptoms. Once again, platelet recruitment (or its absence) appears to be an important point to study in the pathogenesis of the decompression sickness and its resolution (Lambrechts et al., 2018), especially bearing in mind that platelets are increasingly seen as first-line sentinels of the immune system (Sreeramkumar et al., 2014; Hottz et al., 2018).

#### Leukocytes

Even though the initial total number of leukocytes does not seem to be determinant, it appears that a low number of lymphocytes before the dive, also reduced by the minocycline, heralds DCS in the case of an at-risk dive profile. It should be noted that this difference in the lymphocyte count between DCS and NoDCS is no longer true after the dive. This makes it possible to suggest that it is not so much the effective number of lymphocytes present that is important, but rather the number of lymphocytes mobilized. It would also seem that the mobilization of monocytes (macrophage precursors) is preferable in the framework of hyperbaric exposure, insofar as the animals which did not experience a reduction in their number of monocytes following decompression suffered DCS. The recruitment of leukocyte populations, or its absence, could therefore govern the response to the DCS. More globally this idea raises the question of the involvement of the immune system in a seemingly sterile disease, and is potentially the origin of its onset. In the absence of work on which we can really rely, this remains to be demonstrated.

It is interesting to stress that the animals treated with minocycline and destined to present DCS did not mobilize their monocytes or granulocytes significantly, whereas their initial number seems comparable to those of the control NoDCS. Otherwise, a provocative dive inducing the shift of macrophages to the M1 phenotype is described (Han et al., 2017) and also that minocycline disturbs this polarization toward M1 (Kobayashi et al., 2013). The M1 profile can be briefly shown as inflammatory supporting microbiocidal activity but only while M2 macrophages are unable to eliminate pathogens (Mege et al., 2011). In many ways the polarization of monocytes is similar to that of macrophages (Mege et al., 2011). Insofar as many studies (Fink and Cookson, 2005; Kobayashi et al., 2013; Li et al., 2016; Tonnus et al., 2019) associate the inhibitory effect that minocycline may have on caspase-1, NLRP-3 and TLR-4, and the phenotype M1, it should be stressed that the disruption of these pathways seems detrimental in DCS, even though it is necessary to remain prudent about the degree of involvement of this innate immunity pathway in DCS, because its blockage by minocycline did not result in a notable increase in the number of cases of DCS.

### Thermogenesis in DCS Animals

Globally it emerges that DCS mice have a maximum surface temperature which is lower than that of the unscathed mice. More specifically, the processing of the images of the mice tails has made it possible to determine a threshold value for the average temperature, 27.5°C, below which the animals may be considered to be suffering from DCS. Our results show that, whatever the thermal parameter chosen, the DCS mice show temperatures lower than those observed in the NoDCS mice. No effect of minocycline on the temperatures was observed.

The temperature difference observed in the tails between the DCS and NoDCS mice, as well as the increase in the skin surface with a temperature below 27.5°C in the DCS mice, reflects a reduction in the perfusion of the peripheral tissues of the tails of DCS animals or a temperature reduction in the blood irrigating this territory. The reduction in perfusion may be the consequence of vascular obstructions consecutive to platelet aggregation, according to the most common theory about DCS (Brubakk et al., 2003), vasoconstriction, or even a slowing of the heart rate. This deserves further investigation.

As for the reduction in the temperature of the blood irrigating the tail, this would be rather a consequence of a reduction in the internal temperature of the mouse’s body: the Tmax measured by IR is located close to the head or on the head, in the areas where there is no hair, with high blood irrigation and hardly subject to vasoconstriction. The NoDCS mice have a Tmax (36.2; 0.9°C) close to the internal temperature values measured by a rectal probe in animals in the daytime period, i.e., 36.2 ± 1.1°C (Al-Hilli and Wright, 1988). So, if we start from the principle that our Tmax measurement is close to the internal temperature, the drop in Tmax of nearly 3°C in the DCS mice compared with the NoDCS tends to indicate that these DCS mice are in a state of severe hypothermia. This state of hypothermia can alone explain the temperature reduction observed in the tail, also visible in some animals in the hind paws. If this hypothermia state is the consequence of exposure to cold stress suffered in the chamber during decompression, it alone may explain certain of the observations made during the tests performed on leaving the chamber (fatigue, difficulty holding onto the wire during the grip test, etc., ditto perhaps for convulsions). However, this does not explain the differences observed between the DCS and NoDCS mice. The question is, therefore, knowing why the DCS mice have hypothermia.

The environmental conditions are not so restrictive that they exceed the thermoregulation capacities of the NoDCS mice. In fact, they are able to keep their thermal status stable. Another hypothesis for explaining the hypothermia is that the thermoregulatory responses are not correctly put in place. This malfunction may be due to a defect in the regulation chain (incorrect information uptake or non-existent response) which is seen rather as a neurological impairment and/or a defect in the heat production chain with more of an impairment in thermogenesis without shivering produced by the mitochondria in the brown adipose tissue (Morrison et al., 2012). This normally represents the major heat production pathway in mice (Gordon, 2012). Both these systems may be damaged because mitochondrial impairment (Wang et al., 2015) is described in DCS, which may of course explain the neurological impairments as well (Vann et al., 2011). An alternative hypothesis is that hypothermia results from a physiological thermometabolic adaptation, as evoked in works addressing systemic inflammation-associated syndromes (Steiner, 2015; Garami et al., 2018).

The absence of thermal measurements before and just on leaving the chamber do not make it possible to determine whether hypothermia relates to the causes or consequences of DCS. In the absence of rectal temperature measurements, it is also possible that an increase in temperature in the NoDCS mice is under-evaluated due to the inflammation induced by the DCS. Other experiments are required to support these conclusions.

### Limitations

We did not report any beneficial effect for minocycline (90 mg/kg) in this work. For these *in vivo* experiments we administered minocycline orally, without assessing the resulting brain tissue dose. It is therefore possible that the dose of the drug reaching the target tissues, of whatever type, was not high enough to produce significant effects. Indeed, the systemic (i.p.) dose of minocycline required to reach a brain concentration of about 36 μm was reported to be 90 mg/kg for mice (Milane et al., 2007), a dose that is associated with unpleasant side effects such as inflammatory reactions in the peritoneum (Fagan et al., 2004). As, we have chosen to keep the commercialized form (i.e., oral) we must be at the systemic dose of 90 mg/kg, which could explain this lack of therapeutic effect.

An alternative hypothesis to that presented above would be to consider the dose of minocycline used as toxic, given the plasmolytic effects on the red blood cells, and therefore inflammatory. This would result in extra stress on the immune system which would then be less able to deal with this DCS-producing protocol and its resolution. However, as the initial leukocyte counts were similar for the Ctrl and the Mino mice, it does not seem very reasonable to accept this hypothesis.

Extrapolating results from the animal to the human clinic remains an important problem in science. The same goes for the use of infrared imaging. Nevertheless, its use could help to improve animal models where the thermal aspect, however, fundamental, is regularly neglected. In this study, it is likely that we underestimate the cases of DCS, considering the specificity of the test. Although far from a direct clinical application in the absence of further study, it could represent an opportunity -1- to improve ours animal models -2- to check in hyperbaric medicine the perfusion of the cutaneous territories or the presence of bubbles under skin and -3- to help to follow recovery of paralytic patient or neurologic patient (after DCS and hyperbaric treatments) -4- improve monitoring of necrotic wound patient infected with anaerobic bacteria and treated by hyperbaric oxygen. Furthermore, one might also wonder about the effects of anesthetics on thermoregulation, but this point does not seem major since DCS and NoDCS mice were treated the same way, and there was a difference in temperature between the two. In addition, NoDCS mice had a normal temperature.

## Conclusion

Minocycline 90 mg/kg taken early is associated to variation in blood parameters before and after the dive. This seems to limit the mobilization of monocytes and granulocytes after the provocative dive. In this study, a higher proportion of mice treated with minocycline suffered DCS symptoms, but this difference was statistically insignificant. This experiment demonstrates that the role of the immune system in decompression warrants serious investigation, and the interferences with the immune system may be pathogenic when diving. It also emerges that the areas of least blood perfusion have also been observed by infrared imaging. Hypothermia is manifest in the hind paws, the tail and more generally on the maximum temperature reached. Surveillance by infrared camera may be useful to help diagnosis in decompression sickness.

## Data Availability

The raw data supporting the conclusions of this manuscript will be made available by the authors, without undue reservation, to any qualified researcher.

## Ethics Statement

All procedures involving experimental animals were in line with European Union rules (Directive 2010/63/EU) and French law (Decree 2013/118). The Ethics Committee of the Institut de Recherche Biomédicale des Armées approved this study in 2016. According to our Animal Care Committee, a scoring system inspired by Swiss veterinary guidelines was implemented to ensure the welfare of animals. For each animal, a dedicated observer scored the stress or pain (from 0 to 3) relating to specific criteria listed on a form [see, (Cosnard et al., 2017) for more information]. Degree 3 pain (very painful) in one case or a total score of 12 in the table were the ethical endpoints. On this sheet, the most commonly found were: vocalising, aggression or withdrawn behaviour, reduction in exploratory behaviour, licking, closed eyes, tears, bubbles in the eyes, high respiratory rate, runny nose, fur bristling, labored breathing, convulsions, paralysis, difficulty moving and problems with the fore or hind limbs (classified as motor disorders). In this study, no score reached 12 and there was no need to cull the animal based on these criteria. Actually, animals displaying Degree 3 convulsions died very rapidly. At the end of the experiment, rats were anesthetized first with halothane (5% in oxygen, Halothane, Belamont, France) in order to gain time and to minimize stress, and then with an intraperitoneal injection of a mixture of 16 mg/kg xylazine (Rompum^®^ 2%, Bayer Pharma) and 100 mg/kg ketamine (Imalgène^®^ 1000, Laboratoire Rhône). Mice were housed in an accredited animal care facility. Mice were kept in cages (10 per cage) both during rest and during the experiments and maintained on a regular day (6:00 am–6:00 pm)/night (12 h) cycle. Food (kibble from Harlan Laboratories, 18% protein) and water were provided *ad libitum* and the temperature was kept at 22 ± 1°C.

## Author Contributions

A-VD, PL, and NV conceived and designed the research. A-VD, SDM, SG, and NV performed the experiments. A-VD and NV analyzed the data. A-VD, PL, SR, J-EB, SG, SDM, HD, J-JR, and NV interpreted the results of the experiments. NV prepared the figures. A-VD and NV drafted, edited, and revised the manuscript. A-VD and NV approved the final version of the manuscript.

## Conflict of Interest Statement

HD was employed by company Apricot Inhalotherapeutics. The remaining authors declare that the research was conducted in the absence of any commercial or financial relationships that could be construed as a potential conflict of interest.
